# Long-term outcomes of kidney transplantation from expanded criteria donors with Chinese novel donation policy: donation after citizens’ death

**DOI:** 10.1186/s12882-022-02944-y

**Published:** 2022-10-03

**Authors:** Xiao Fang, Yan Wang, Rong Liu, Changyan Zhu, Chenguang Wu, Fuqiang He, Shunliang Yang, Dong Wang

**Affiliations:** 1 Department of Urology, 900th Hospital of the Joint Logistics Team, No.156 Xi’erhuan North Road, Fuzhou, 350025 China; 2 Department of Urology, MengChao Hepatobiliary Hospital of Fujian Medical University, No.312 Xihong Road, Fuzhou, 350001 China; 3 Department of Nephrology, MengChao Hepatobiliary Hospital of Fujian Medical University, No.312 Xihong Road, Fuzhou, 350001 China

**Keywords:** Kidney transplantation, Donation after Citizens’ Death, Expended criteria donor, Outcomes

## Abstract

**Introduction:**

The Chinese Government initiated the Donation after Citizens' Death policy in 2010. To now, it has been a major source of organs for transplant. Since it is still a young policy, corresponding clinical evidence is still urgently needed for its improvement. Compared to kidneys donated by SCD (standard criteria donor), increasing the use of ECD (expanded criteria donor) derived kidneys is a way to expand the donor pool but is also a result of the aging demography of China. This study is based on the data of kidney transplantation in our center with the Donation after Citizens' Death policy, aiming to provide a reference for the clinical use of ECD kidneys.

**Method:**

A retrospective study enrolled 415 kidney transplants derived from 211 donors performed between October 2011 and October 2019. A total of 311 (74.9%) organs were donated from 159 (75.4%) SCDs, and the remaining 104 (25.1%) were from 52 (24.6%) ECDs. The log-rank test was used to compare the difference in survival and postoperative complications. The Chi-square test was used to compare the occurrence of postoperative complications and postoperative renal function. The Cox regression analysis was used for risk factor screening.

**Result:**

Analysis showed that grafts from ECD were poorer in survival (*P* = 0.013), while their recipients had comparable (*P* = 0.16) survival. Moreover, it also was an independent risk factor for graft loss (HR 2.27, *P* = 0.044). There were significantly more AR occurrences in the ECD group compared with SCD group (25.0% vs. 15.8%, *P* = 0.004), but no significant difference was found in infection (51.9% vs. 47.6%, *P* = 0.497) and DGF (26.0% vs. 21.9%, *P* = 0.419) between them. Similarly, fewer recipients in the ECD group were free from AR within 1 year after transplantation (*P* = 0.040), with no statistical difference in all-cause infection prevalence in 1 year (*P* = 0.168). The eGFR in the ECD group was significantly worse than that in the SCD group at 3 months, 6 months, 1 year, 3 years, and the highest value posttransplant (all < 0.05), but no difference at 5 years posttransplant. Besides, results showed cardiac arrest (uncontrolled vs. controlled, HR 2.49, *P* = 0.049), HLA mismatch (4–6 loci vs. 0–3 loci, HR 3.61, *P* = 0.039), and AR occurrence (HR 2.91, *P* = 0.006) were demonstrated to be independent risk factors for graft loss.

**Conclusion:**

The ECD-derived kidney was worse than the SCD-derived kidney in terms of graft survival and AR occurrence, and trend to an inferior renal function postoperative. However, the recipient survival, DGF occurrence, and all-cause infection occurrence were similar.

## Introduction

Even though kidney transplantation is the best treatment for end-stage renal disease, the severe shortage of transplantable organs remains an unavoidable topic [1, 2]. With the aging demography of China, the number of potential elder donors who die of hypertension and cerebrovascular accidents also increases. As opposed to the kidneys from standard criteria donor (SCD), the clinical use of expanded criteria donor (ECD) derived kidneys was getting more focus recently [3].

The Chinese Government initiated the Donation after Citizens' Death policy in 2010, which was then promoted nationwide after a 3-year pilot implementation period [4]. To now, it has been a major source of organs for transplant. Since it is still a young policy, there are differences in the mature international practices regarding recipients' race, organ procuring, distribution rules, organ function quality assessment, perioperative management of recipients, etc. [5]. Corresponding clinical evidence is still urgently needed for its improvement.

This study is based on the data of kidney transplantation in our center with the Donation after Citizens' Death policy to compare the long-term outcomes of ECD and SCD organs in multi-aspects, aiming to provide a reference for the clinical use of ECD kidneys.

## Materials and methods

### Patients and study design

This retrospective study enrolled 415 kidney transplants from 211 donors based on the Donation after Citizens’ Death conducted between October 2011 and October 2019. The ECD meets that the donor is older than 60 years old, or between 50 and 59 years old, and meets at least two of the following criteria: 1. Final serum creatinine > 1.5 mg/dL(132 μmol/L), 2. Cerebrovascular accident as the cause of death, 3. History of hypertension [3]. Based on this criterion, the donors, corresponding kidneys, and corresponding recipients were divided into the ECD and SCD groups for analysis.

All patients awaiting kidney transplants with end-stage renal disease were registered in the China Organ Transplant Response System (COTRS). Patients with contraindications for kidney transplantation (such as metastatic malignancy, active presence of HIV infection, and other reasons) were excluded. The CORTS algorithm was rigorously adhered to. Higher panel-reactive antibody (PRA) levels led to a lower priority for transplant, and we avoided transplantation in patients with PRA > 30%. To lower the rate of rejection following transplantation, prophylactic therapies (such as plasmapheresis, immunoadsorption, and medication therapy) were administered to enrolled patients with high immunogenicity (peak PRA > 50%).

The donor family members (spouses, adult children, and parents) consented to organ donation after death and signed the appropriate informed consent paperwork voluntarily. Donors who did not meet the usage criteria based on a needle biopsy performed prior to transplantation were ruled out [6].

Data were collected from the electronic medical record system and the registry system of the organ donation database of our center, which were analyzed anonymously. Following surgery, recipients were intensively monitored during the hospitalization and then followed up by the out-patient clinic at regular intervals after discharge. Table 1 shows the patient features.

**Table1:** Baseline characteristics of donors and recipients

	**ECD**	**SCD**	***P* value**
**Donors**	***N* = 52**	***N* = 159**	
Age (years)	54.31 ± 3.96	30.99 ± 11.84	< 0.001
Sex, n (%)			0.652
Male	46 (88.5%)	134 (84.3%)	
Female	6 (11.5%)	25 (15.7%)	
BMI (kg/m^2^)	22.7 (21.2–24.2)	22.0 (20.2–23.4)	0.061
History of hypertension, n (%)	38 (73.1%)	20 (12.6%)	< 0.001
HBV infection, n (%)	25 (15.7%)	7 (13.5%)	0.825
Cause of death, n (%)			0.001
Cerebrovascular accident	29 (55.8%)	44 (27.7%)	
Trauma	19 (36.5%)	99 (62.3%)	
Other	4 (7.7%)	16 (10.1%)	
Terminal Scr (μmol/L)	134.0 (63.9–165.6)	96.0 (67.0–142.8)	0.160
**Recipients**	***N* = 104**	***N* = 311**	
Age (years)	40.04 ± 11.03	38.53 ± 10.09	0.197
Sex, n (%)			0.715
Male	73 (70.2%)	210 (67.5%)	
Female	31 (29.8%)	101 (32.5%)	
BMI (kg/m^2^)	21.2(19.2–23.0)	21.1 (18.8–23.0)	0.382
Cause of renal failure, n(%)			0.446
Glomerulonephropathy	80 (76.8%)	219 (70.4%)	
IgA nephropathy	8 (7.7%)	38 (12.2%)	
Diabetic nephropathy	8 (7.7%)	21 (6.8%)	
Others	8 (7.7%)	33 (10.6%)	
History of hypertension, n (%)	59 (56.7%)	158 (50.8%)	0.309
History of diabetes, n (%)	25 (24.0%)	53 (17.0%)	0.146
Dialysis duration (months)	24.0 (12.0–39.8)	18.0 (10.0–36.0)	0.058
HLA mismatches			0.135
0–3	37 (35.6%)	85 (27.3%)	
4–6	67 (64.6%)	226 (72.7%)	
Cold ischemia time (h)	8.94 ± 2.70	8.0 ± 2.69	0.974
Negative PRA, n (%)	98 (94.2%)	296 (95.2%)	0.792
Remuzzi score	3.07 ± 1.14	2.82 ± 1.26	0.076
**Process**			
Cardiac arrest, n (%)			0.395
Controlled	46 (88.5%)	147 (92.5%)	
Uncontrolled	6 (11.5%)	12 (7.5%)	
Warm ischemia time (min)			0.631
≤ 15 min	26 (50.0%)	72 (45.3%)	
> 15 min	26 (50.0%)	87 (54.7%)	
Induction therapy, n (%)			0.603
ATG	25 (8.0%)	10(9.6%)	
Basiliximab	263(84.6%)	89(85.6%)	
Others	23(7.4%)	5(4.8%)	

### Immunosensitivity test

Before kidney transplantation, three pairs of the six human leukocyte antigens (HLA) –A, -B, and –DR were tested. Crossmatch testing for complement-dependent cytotoxicity (CDC) was negative in all recipients. PRA of recipients was routinely tested before transplantation by an enzyme-linked immunosorbent assay (ELISA) including PRA –I and PRA-II. We defined peak PRA < 10% as negative.

### Immunosuppression protocol

Induction therapy mainly consisted of anti-interleukin-2 receptor monoclonal antibody (basiliximab, Simulect®, Novartis) or anti-thymocyte globulin (ATG, Thymoglobuline®, Genzyme). Patients without the HLA antibodies received basiliximab, which was administered in two 20 mg doses by bolus intravenous injection. The first bolus was given within the 2 h before revascularization of the graft and the second one is on day 4 post-transplant. Patients with HLA antibodies were given single bolus ATG induction therapy at a dose of 50-75 mg. ATG was regular intravenous infusion within 6 h before graft’s revascularization and maintain 25 mg daily until 3 days post-transplant. Before starting induction therapy, 40 mg of methylprednisolone was injected intravenously to prevent the side effects of ATG and basiliximab.

Standard immunosuppressive triple therapy consists of tacrolimus (FK-506) or ciclosporin A (CsA), mycophenolate mofetil (MMF) or mycophenolate sodium (MPS), and prednisone. MMF (1 to 2 g/day) or MPS (0.72 to 1.44 g/day) was administered immediately following the transplant. The dosage was adjusted based on the blood routine examination of patients and tapered over time. The administration of CsA (6 ~ 8 mg/kg/day) or FK-506 (0.1 ~ 0.15 mg/kg/day) was initiated from day 1 of post-transplant, and the doses were adjusted according to the trough levels of the drugs.

The serum concentrations of FK-506 and CsA were routinely monitored following kidney transplantation. For FK-506, serum was obtained half an hour prior to administration (C_0_), and for CsA, serum was collected at C_0_ and 2 h following medication (C_2_).

The target levels were timely varying at 1 month, 1 to 3 months, 4 to 12 months, and > 1 year following transplantation. The C_0_ target levels of FK-506 concentrations were decreased from 8–12 ng/mL to 6–10 ng/mL, 4–10 ng/mL and 4–8 ng/mL. The C_0_ target levels of CsA reduced from 150–300 ng/mL to 150–250 ng/mL, 120–250 ng/mL and 80–120 ng/mL. The C2 target levels of CsA concentrations were reduced from 1000–1500 ng/mL to 800–1200 ng/mL, 600–1000 ng/mL and > 400 ng/mL, respectively. The target level was also individually modified according to the patient’s condition. Oral MMF (1 to 2 g/day) or MPS (0.72 to 1.44 g/day) also continued to be used for maintenance immunosuppressive therapy and individually modified according to the patient’s conditions. Oral prednisone was subsequently prescribed at a daily dose of 20 mg. Then the daily dose was tapered to 10 mg in 6 months.

### Definitions

Delayed graft function (DGF) is used to describe the status of transplanted kidneys that fail to function immediately after transplantation and is a significant complication of kidney transplantation. In this study, DGF was defined as the need for dialysis during the first week after transplantation [7].

Acute rejection (AR) was suggested clinically by an unexplained rise in serum creatinine concentration of > 0.3 mg/dL or a 25% increase from baseline [8]. The diagnosis of AR was confirmed by percutaneous kidney biopsy, and kidney pathology was classified using Banff 07 classification and its subsequent updates [9].

Warm ischemia time (WIT) was defined as the time interval between the withdrawal of life support to cold perfusion.

The all-cause infections in 1-year post-transplant were also analyzed, including surgical site infection, and pulmonary, and urinary tract infections.

The estimated glomerular filtration rate (eGFR) was calculated by using the CKD-EPI eGFR equation based on gender, age, and serum creatine (Scr) [10].

### Statistical analysis

Results were expressed as percentages for categorical variables and numerical values for continuous variables, respectively. The baseline features of the SCD and ECD groups were compared using the Chi-square test, Mann–Whitney U test, and Student’s t-test as appropriate. The Kaplan–Meier method and Log-rank test were used to compare the graft/recipient survival, occurrence of AR and all-cause infection between the two groups and generated the survival curve. The log-rank test was used to analyze statistical differences between curves. Differences in the incidence of DGF between the two groups were determined by using the Chi-square test. Comparisons of eGFR were using the Mann–Whitney U test. Cox univariate/multivariate regression analysis was utilized to determine risk factors and the hazard ratio for graft failure. Every test was two-tailed. *P* values < 0.05 were regarded as statistically significant.

## Result

### Baseline characteristics of patients

In this retrospective study, we included and analyzed 211 donors and their corresponding 415 donated kidneys, of which 7 kidneys were discarded because the needle biopsy results did not meet the criteria for use. The kidney utilization rate is 98.3%. A total of 311 (74.9%) organs were donated from 159 (75.4%) SCDs, and the remaining 104 (25.1%) were from 52 (24.6%) ECDs. The median follow-up period of this study was 1069 (range from 147 to 3074) days. The comparisons of baseline characteristics between the ECD group and the SCD group are shown in detail in Table 1. The mean age and prevalence of hypertension in ECDs were significantly higher than in SCDs (*p* < 0.001). No other significant difference was observed for other characteristics between the two groups.

### Graft and recipient outcomes

**Fig. 1:**
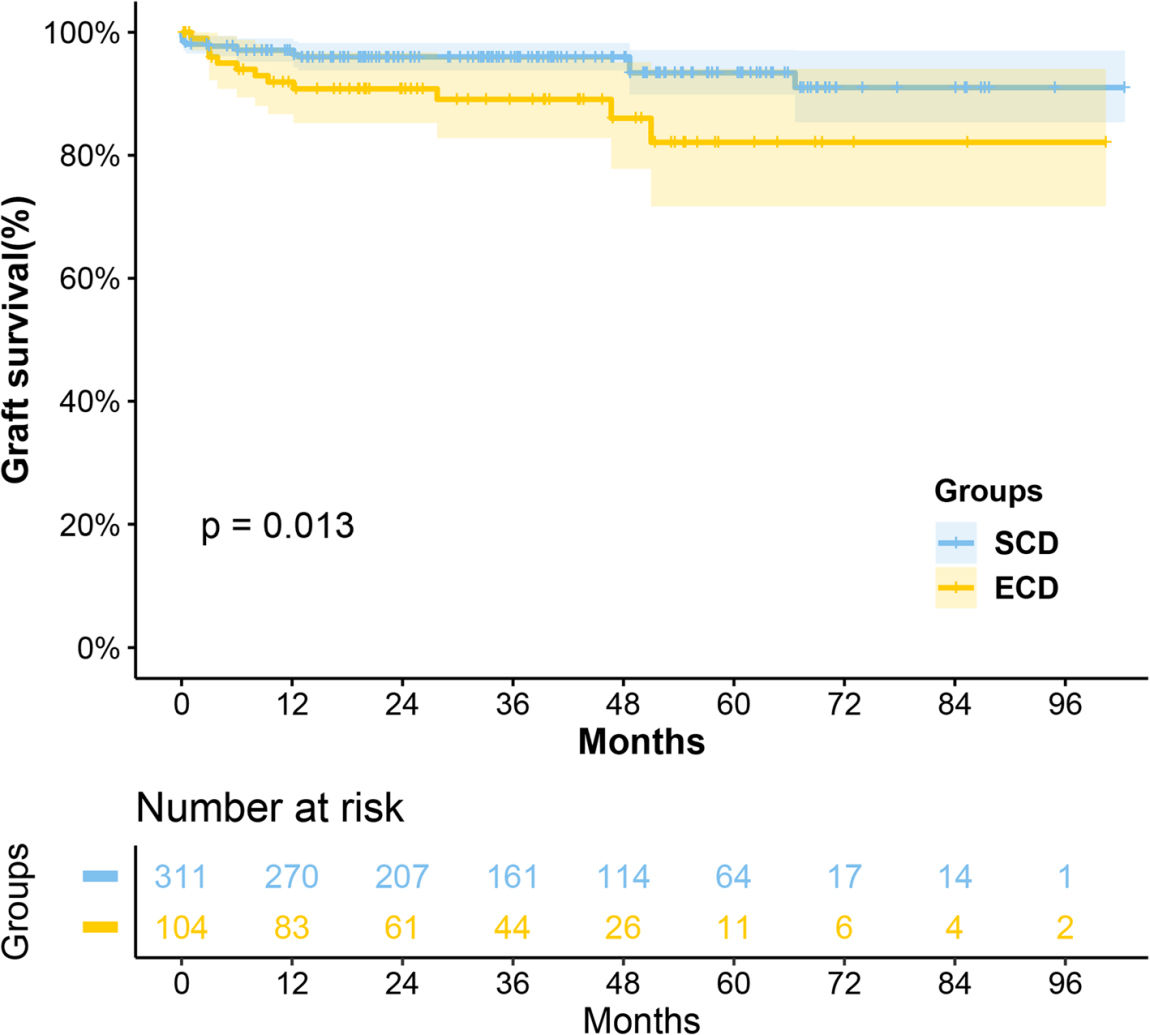
Graft survival curves for the ECD group and the SCD group

**Fig. 2:**
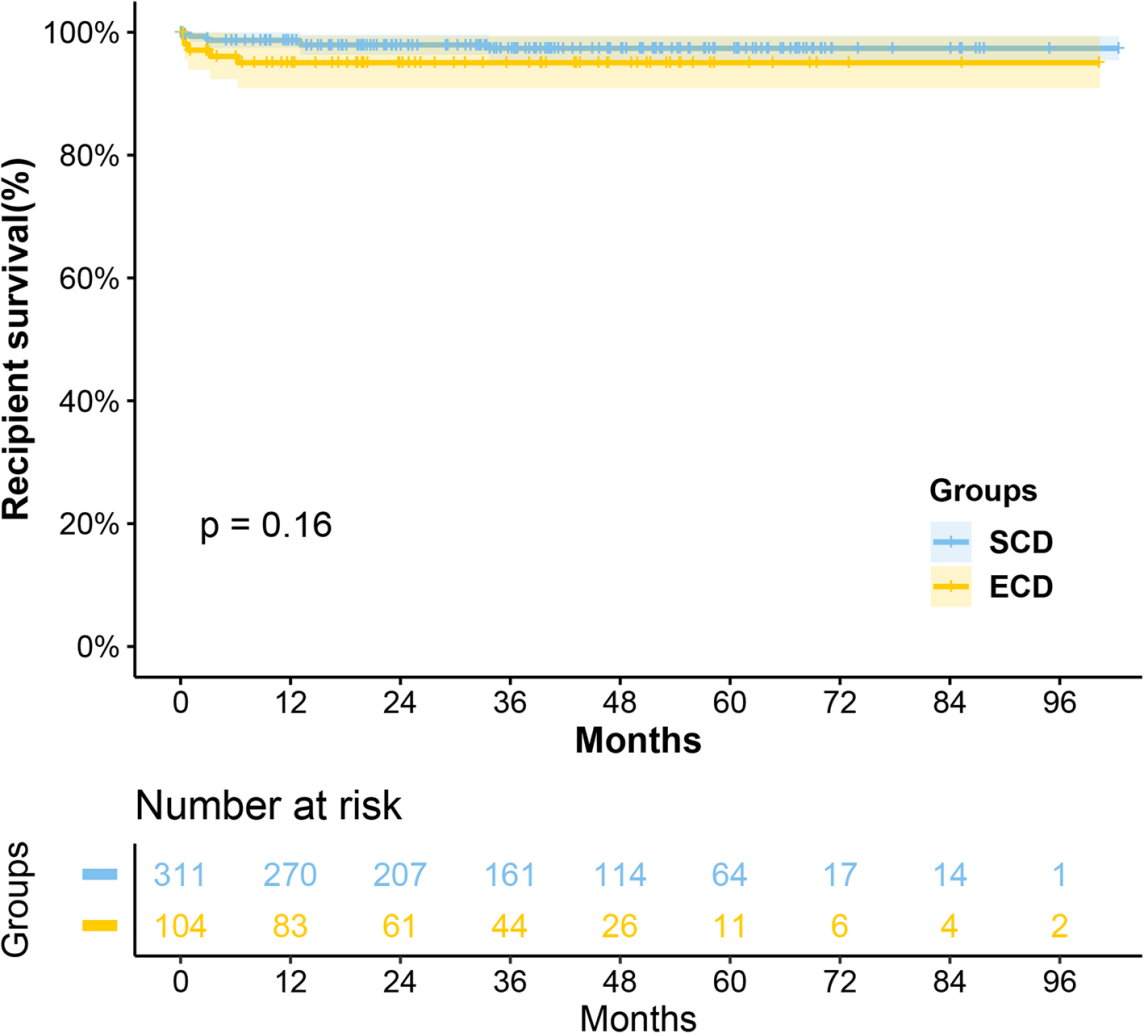
Recipient survival curves for the ECD group and the SCD group

In this study, we analyzed graft survival and recipient survival separately. There were 12 deaths of recipients and 40 grafts lost during the follow-up period. No primary non-function case was observed. There were 15(3.6%) cases of early graft loss (loss within 90 days after transplantation). Among all cases of graft failure, the most common causes were rejection (13, 32.5%), infection (9, 22.5%), and graft hemorrhage (8, 20.0%). The 1-, 3-, 5-years of graft survival rates in the ECD group were 86.3%, 84.7%, and 78.0%, while those in the SCD group were 95.1%, 93.5%, and 91.0%. The 1-, 3-, and 5-years recipient survival rates were consistently at 95.0% in the ECD group, while in the SCD group, the recipient survival rates were 98.7%, 97.4%, and 97.4%, respectively. According to the log-rank test, kidneys from ECD had poorer survival in the early period after transplantation (*P* = 0.001 in the first year, *P* = 0.011 in the second year, and *P* = 0.007 in the third year), and this disparity continued throughout our follow-up period (Fig. 1, *P* = 0.013), while except for the first year, their recipients had comparable long-term survival (*P* = 0.032 in the first year, *P* = 0.105 in the second year, *P* = 0.156 in the third year, and allover *P* = 0.16, Fig. 2).

### Post-transplant complications

The occurrence of complications post-transplant, including AR, all-cause infection, and DGF, has shown in Table 2. After transplantation, there were significantly more AR occurrences in the ECD group (25.0% vs. 15.8%, *P* = 0.004). Nevertheless, no significant difference was found in infection (51.9% vs. 47.6%, *P* = 0.497) and DGF (20.2% vs. 18.3%, *P* = 0.666) between ECD group and SCD group.

**Table 2:** Post-transplant complications in the ECD and SCD groups

	ECD	SCD	*P* value
1-year free from acute rejection ^a^	80.8%	90.6%	0.004
Acute rejection ^b^	26 (25.0%)	49 (15.8%)	0.040
1-year free from all-cause infection ^a^	72.1%	77.8%	0.168
All-cause infection ^b^	54 (51.9%)	148 (47.6%)	0.497
Delayed graft function ^b^, n (%)	21 (20.2%)	57(18.3%)	0.666

^a^ The Kaplan–Meier method was performed to compare outcomes in the two groups, and the log-rank test was performed to identify the difference between the two groups. Outcomes were reported as frequencies of freedom from events in 1-year post-transplant

^b^ The Chi-square test was performed to identify the difference between the two groups. Outcomes were reported as numbers (percentages) of events during the follow-up period

**Fig. 3:**
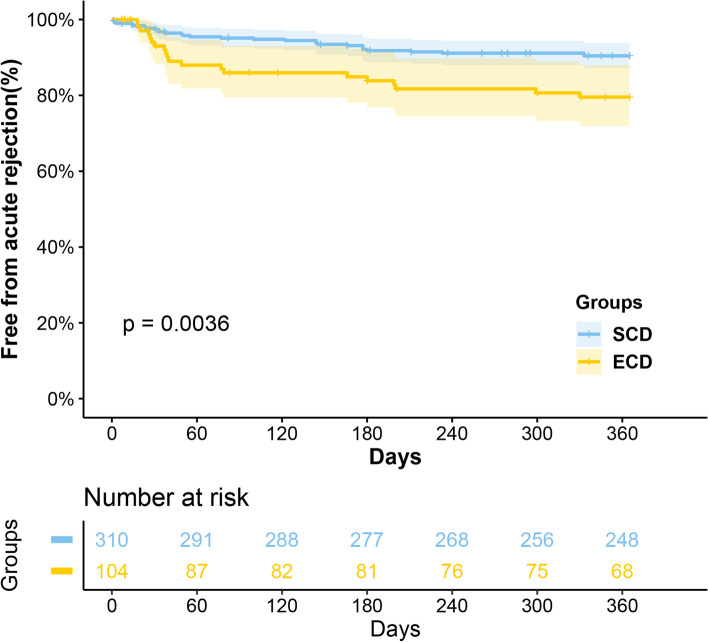
Curves for the 1-year freedom of acute rejection by groups

**Fig. 4:**
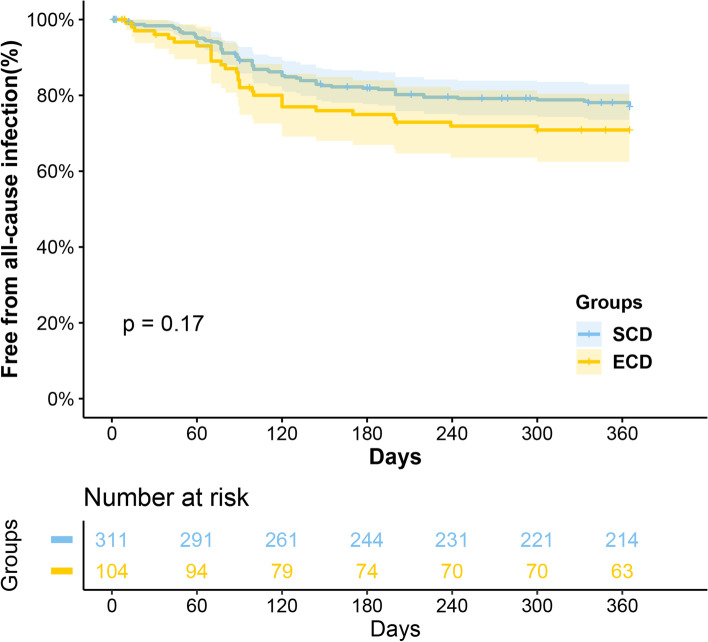
Curves for the 1-year freedom of all-cause infection by groups

Since AR and infection may occur several times, and the incidence is higher within 1 year after transplantation, we analyzed their incidence that did not occur within 1 year by the Kaplan–Meier method and Log-rank test (Figs. 3 and 4). Similarly, we found that fewer recipients in the ECD group were free from AR within 1 year after transplantation (*P* = 0.040), while no statistical difference was found in all-cause infection prevalence (*P* = 0.168).

### Evaluation of the therapeutic effect

As a main post-transplant efficacy parameter, eGFR was calculated with the CKD-EPI equation and compared by groups (Table 3). We found that the eGFR of ECD donor kidneys was significantly lower than that of SCD donor kidneys in the early and medium-term after transplantation (*P* < 0.001 at 3 months, 6 months, and 1 year, *P* = 0.002 at 3 years), while eGFR tended to be similar at 5 years (*P* = 0.502). In addition, the highest eGFR in the SCD group was better than that in the ECD group (*P* < 0.001).

**Table 3:** Comparison of eGFR between the two groups at different follow-up time points after transplantation^a^

Time	ECD group		SCD group		*P*-value
	No	eGFR^b^	No	eGFR^b^	
3-month	98	53.8(41.4–66.7)	302	65.9(54.0–80.0)	< 0.001
6-month	94	52.1(43.1–69.7)	298	69.1(55.0–81.8)	< 0.001
1-year	83	59.1(41.6–71.8)	269	72.0(56.6–82.5)	< 0.001
3-year	44	62.7(48.5–75.9)	158	73.6(58.7–91.6)	0.002
5-year	11	79.5(74.7–86.4)	58	81.7(65.1–98.0)	0.502
Highest^c^	103	53.7(22.1–78.5)	307	72.2(52.4–91.1)	< 0.001

^a^ Comparison of the two groups was using the Mann–Whitney U test

^b^ Estimated glomerular filtration rate was calculated using the CKD-EPI eGFR_Scr_ equation and was in units of ml/min/1.73m^2^

^c^ Excluded cases with early graft loss before eGFR turned normal (4 cases in SCD group and 1 case in ECD group)

### Risk factors for graft survival

**Fig. 5:**
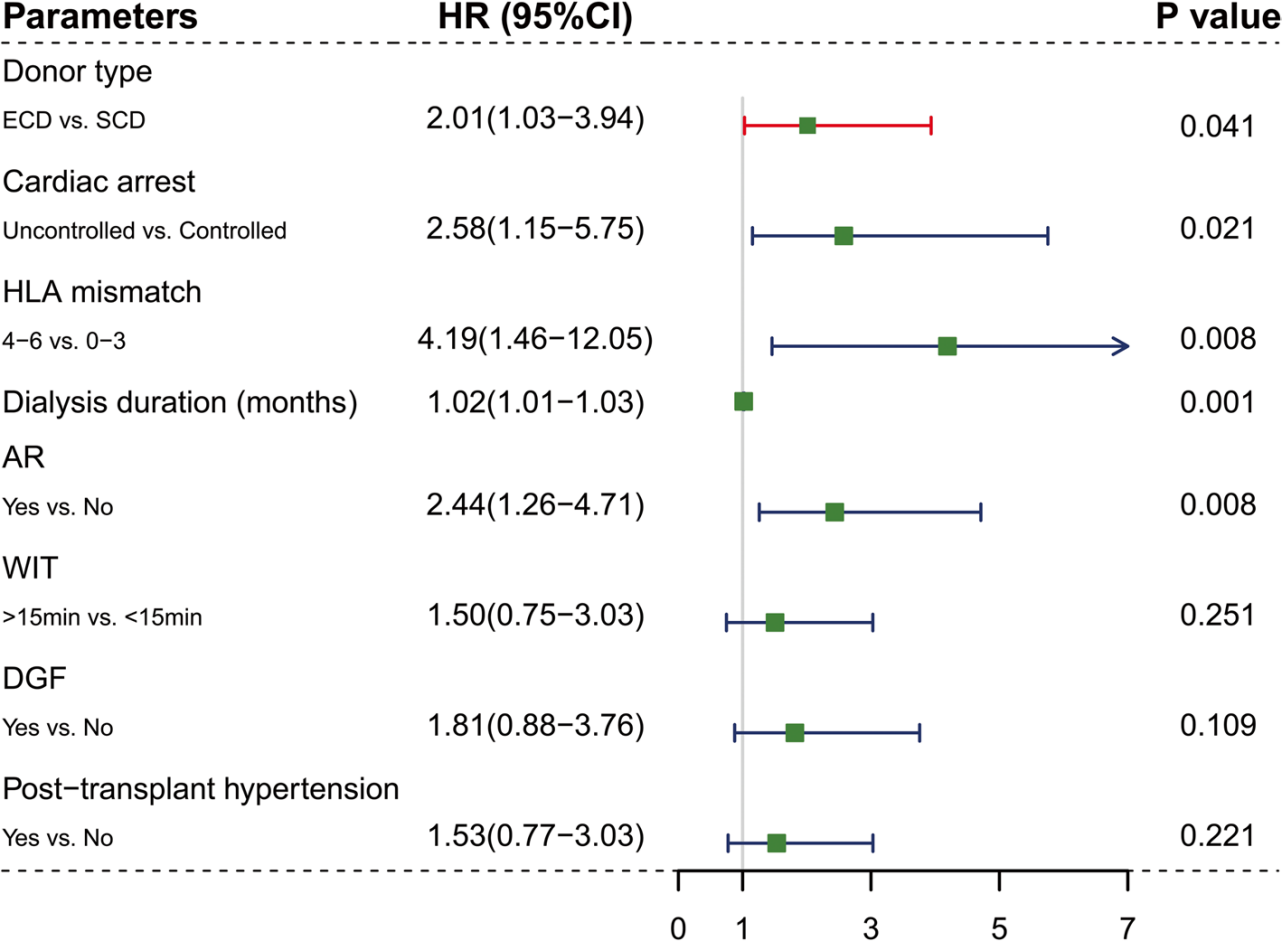
Multivariate analysis to identify risk factors and hazard ratio for death-censored graft survival. ^a^. ^a^ Cox hazard ratio model was performed to identify the factors influencing graft survival

The univariate analysis showed that donors' age, donors' Body Mass Index (BMI), donors' terminal Scr, recipients' age, recipients' BMI, recipient history of diabetes, graft volume, HBV infection, and cold ischemia time (CIT) were not prognostic factors for the graft loss (Table 4). Multivariate analysis revealed 5 independent risk factors for long-term graft loss. In addition to donor type (ECD vs. SCD, HR 2.01, *P* = 0.041), which is the focus of this study, cardiac arrest (uncontrolled vs. controlled, HR 2.58, *P* = 0.021), HLA mismatch (4–6 loci vs. 0–3 loci, HR 4.19, *P* = 0.008), AR occurrence (HR 2.44, *P* = 0.008), and prolonged dialysis duration(HR 1.02, *P* = 0.001) were also statistically significant (Fig. 5).

**Table 4:** Univariate analysis of risk factors for graft survival (non-significant)^a^

	Hazard ratio	95% CI	*P* value
Donors’ age (year)	1.018	0.991–1.045	0.184
Donnors’ BMI (kg/m^2^)	0.960	0.857–1.075	0.480
Donors’ terminal Scr (μmol/L)	1.001	0.996–1.006	0.646
Recipients’ age (year)	1.009	0.973–1.045	0.639
Recipients’ BMI (kg/m^2^)	0.997	0.973–1.022	0.831
Recipient history of diabetes (yes vs. no)	1.677	0.783–3.807	0.217
Graft volume (mL)	1.000	0.996–1.005	0.945
HBV infection (yes vs. no)	0.478	0.113–2.014	0.314
Cold ischemia time (h)	1.091	0.972–1.226	0.140

^a^ Cox hazard ratio model was performed to identify the negative factors influencing graft survival

## Discussion

Due to the severe imbalance between the supply and demand of transplantable kidneys, some patients remain on dialysis and experience long waits on the waitlist. In 2020, only about a quarter of waitlisted patients received a deceased donated kidney transplant within 5 years [1]. While the Donation after Citizens' Death policy becomes an effective way to address this urgent need [4]. As one of the pilot centers, our center has performed kidney transplantation with this new policy since 2011. However, there are few long-term studies on it due to its short implementation period. There are still many rules that need to be reconsidered and improved. Corresponding long-term clinical evidence is needed. Despite the potential risks associated with ECD-derived organ use, the clinical benefits cannot be denied [11]. Increasing the use of ECD-derived kidneys is a way to expand the donor pool but is also a result of the aging demography of China. So, evaluating and using ECD-derived kidneys is of great significance.

This study presented the results of 415 kidney transplants at our center since the implementation of the Donation after Citizens' Death policy. Its primary outcomes were satisfying and comparable to established international practices [12]. This study used the conventional definition of ECD, which makes the result comparable with results published internationally. Previous studies have shown poor survival in ECD-derived kidneys [13–15], as demonstrated in our study (Log-rank test, *P* = 0.013; Multivariate cox regression, *P* = 0.044, HR = 2.27). However, the results of this study were slightly higher than those of similar studies in terms of patient and graft survival. This may be because our center is cautious in the selection of donated kidneys, and the age of both donors and recipients (especially the ECD group) is slightly lower than that of them, leading to a better prognosis. In addition, in the results of this study, the eGFR of ECD-derived kidney recipients was significantly worse than that of SCD-derived kidney recipients at 3 months, 6 months, 1 year, 3 years, and the highest value posttransplant. That has a similar result to the cohort from Nagaraja et al. [14]. The statistical difference in eGFR turned negative at 5 years after transplantation may be due to the tendency of kidney loss with poor renal function.

The results of postoperative complications in this study showed that ECD-derived kidney recipients were more frequent to develop AR after surgery, while there was no difference in DGF and all-cause infection. Similar results can be observed in other clinical practices [16, 17]. The overall age of the recipients in this study was 40.04 ± 11.03 years old, a young group. In organ transplantation practice, young recipients have a more robust immune response to antigens. While due to proper T-cell effector immune response with an intact regulatory and memory T-cell response, the aged recipients may be weaker immune responses [18]. A recent study by Iske et al. [19] found that the increased content of free mitochondrial DNA in the organs of elderly donors would activate CD11c and DC cells of the recipients, thereby promoting the proliferation of helper T cells and the secretion of IL-17A. This immune response was more robust in young recipients and more likely to lead to AR occurrence. This may also be one of the mechanisms contributing to this result.

The value of ECD-derived kidney clinical use is still controversial [20]. A study comparing ECD recipients with dialysis patients published in JAMA showed that only diabetic recipients and candidates older than 40 years with long waiting times could benefit from receiving an ECD donor kidney [11]. Thus, ECD-derived kidneys may provide a benefit, but it is limited. So, finding an appropriate way to use and allocate these marginal kidneys became a tremendously meaningful topic. At present, the mainstream view prefers to allocate ECD-derived kidneys to older recipients. Since 1999, European countries have recommended the implementation of the European Advanced Transplant Program, a so-called "old-to-old" allocation system, so that kidneys from donors over 65 years old prefer to be allocated to recipients over 65 years old to reduce the wait time for organs [21]. Similarly, In 2014, a new kidney allocation system was implemented in the United States. They expanded the donation criteria and included matching the recipients' life expectancy with organ life expectancy in allocate rules. In this way, the donor pool is expanded, and more elderly patients have access to organ transplants [22]. Based on the above references, the allocation rules of the Donation after Citizens' Death policy for organs from the elderly donor can be reconsidered and further defined.

This study also showed that the occurrence of AR, more mismatches HLA loci, and uncontrolled cardiac arrest, prolonged dialysis duration as predictors of graft loss. Among them, AR occurrence and more HLA mismatch may cause immunogenic injury, uncontrolled cardiac arrest leads to longer ischemia time and ischemia–reperfusion injury, the adverse effects of the prolonged period of dialysis on kidney graft survival have been demonstrated in clinical studies [4, 23]. These risk factors had been well explored in clinical research, and results could guide clinical work.

There were still several limitations to our study. Some factors have been confirmed to be independent risk factors for kidney allograft loss (such as DGF occurrence [24] et al.), and no significant results were obtained in our study, which may be due to the statistical bias caused by median sample size. Besides, inclusion bias and omitted confounding factors may also influence the results.

## Conclusion

The ECD-derived kidney was worse than the SCD-derived kidney in terms of graft survival and AR occurrence, and trend to an inferior renal function postoperative. However, the recipient survival, DGF occurrence, and all-cause infection occurrence were similar. The findings of this study provide evidence for the clinical use of ECD-derived kidneys and improve organ procurement policy in China.

## Data Availability

The datasets used and/or analysed during the current study available from the corresponding author on reasonable request.

## Abbreviations

AR: Acute rejection;
BMI: Body Mass Index
CDC: Complement-dependent cytotoxicity
COTRS: China Organ Transplant Response System
CsA: Ciclosporin A
CIT: Cold ischemia time
DGF: Delayed graft function
ECD: Expanded criteria donor
ELISA: Enzyme-linked immunosorbent assay
eGFR: Estimated glomerular filtration rate
FK-506: Tacrolimus
MMF: Mycophenolate mofetil
MPS: Mycophenolate sodium
PRA: Panel-reactive antibody
SCD: Standard criteria donor
Scr: Serum creatine
HLA: Human leukocyte antigen
WIT: Warm ischemia time
